# Association of Physician Burnout With Suicidal Ideation and Medical Errors

**DOI:** 10.1001/jamanetworkopen.2020.28780

**Published:** 2020-12-09

**Authors:** Nikitha K. Menon, Tait D. Shanafelt, Christine A. Sinsky, Mark Linzer, Lindsey Carlasare, Keri J. S. Brady, Martin J. Stillman, Mickey T. Trockel

**Affiliations:** 1 Department of Psychiatry and Behavioral Sciences, Stanford University School of Medicine, Stanford, California; 2 Department of Medicine, Stanford University School of Medicine, Stanford, California; 3 Professional Satisfaction and Practice Sustainability, American Medical Association, Chicago, Illinois; 4 Department of Medicine, Hennepin Healthcare, Minneapolis, Minnesota; 5 Department of Health Law, Policy & Management, Boston University School of Public Health, Boston, Massachusetts

## Abstract

**Importance:**

Addressing physician suicide requires understanding its association with possible risk factors such as burnout and depression.

**Objective:**

To assess the association between burnout and suicidal ideation after adjusting for depression and the association of burnout and depression with self-reported medical errors.

**Design, Setting, and Participants:**

This cross-sectional study was conducted from November 12, 2018, to February 15, 2019. Attending and postgraduate trainee physicians randomly sampled from the American Medical Association Physician Masterfile were emailed invitations to complete an online survey in waves until a convenience sample of more than 1200 practicing physicians agreed to participate.

**Main Outcomes and Measures:**

The primary outcome was the association of burnout with suicidal ideation after adjustment for depression. The secondary outcome was the association of burnout and depression with self-reported medical errors. Burnout, depression, suicidal ideation, and medical errors were measured using subscales of the Stanford Professional Fulfillment Index, Maslach Burnout Inventory–Human Services Survey for Medical Personnel, and Mini-Z burnout survey and the Patient-Reported Outcomes Measurement Information System depression Short Form. Associations were evaluated using multivariable regression models.

**Results:**

Of the 1354 respondents, 893 (66.0%) were White, 1268 (93.6%) were non-Hispanic, 762 (56.3%) were men, 912 (67.4%) were non–primary care physicians, 934 (69.0%) were attending physicians, and 824 (60.9%) were younger than 45 years. Each SD-unit increase in burnout was associated with 85% increased odds of suicidal ideation (odds ratio [OR], 1.85; 95% CI, 1.47-2.31). After adjusting for depression, there was no longer an association (OR, 0.85; 95% CI, 0.63-1.17). In the adjusted model, each SD-unit increase in depression was associated with 202% increased odds of suicidal ideation (OR, 3.02; 95% CI, 2.30-3.95). In the adjusted model for self-reported medical errors, each SD-unit increase in burnout was associated with an increase in self-reported medical errors (OR, 1.48; 95% CI, 1.28-1.71), whereas depression was not associated with self-reported medical errors (OR, 1.01; 95% CI, 0.88-1.16).

**Conclusions and Relevance:**

The results of this cross-sectional study suggest that depression but not physician burnout is directly associated with suicidal ideation. Burnout was associated with self-reported medical errors. Future investigation might examine whether burnout represents an upstream intervention target to prevent suicidal ideation by preventing depression.

## Key Points

**Question:**

Is burnout associated with increased suicidal ideation and self-reported medical errors among physicians after accounting for depression?

**Findings:**

In this cross-sectional study of 1354 US physicians, burnout was significantly associated with increased odds of suicidal ideation before but not after adjusting for depression and with increased odds of self-reported medical errors before and after adjusting for depression. In adjusted models, depression was significantly associated with increased odds of suicidal ideation but not self-reported medical errors.

**Meaning:**

The findings suggest that depression but not burnout is directly associated with suicidal ideation among physicians.

## Teaser

This cross-sectional study uses data from surveys completed by US physicians to examine associations between physician burnout, suicidal ideation, and self-reported medical errors after adjusting for depression.

## Introduction

Studies have shown that a career in medicine is associated with increased risk of suicide.^1,2,3,4,5,6^ A recent analysis^7^ suggests that the increased risk for suicide among attending physicians may be declining or previously overestimated. However, reports among physicians in training indicate an association with increased risk for suicide^1,2,3^ despite certain risk factors associated with suicide in the general population (eg, financial constraints, job security) being uncommon in medicine. Approximately 1 in 10 medical students,^1^ 1 in 4 interns,^8^ and 1 in 16 practicing physicians^9^ report some degree of suicidal ideation. Although medical students exhibit better mental health indexes than do age-matched peers at matriculation, these measures decline during the course of their education.^2^

Depression, substance abuse, impaired relationships, self-destructive tendency, and guilty self-concept^10^ are associated with physician suicide.^7^ Suicidal ideation has also been associated with occupation-specific factors, including practicing psychiatry or anesthesiology,^7^ increased workload volume,^7^ being evaluated as unfit to practice,^11^ perceived medical errors by surgeons,^9^ workplace harassment, and lack of empowering leadership among postgraduate physicians in training.^12^ Previous research also identifies burnout—an occupational syndrome recognized by the World Health Organization^13^ and experienced by physicians at epidemic levels^14,15^—as a factor associated with both depression and suicide in physicians and physicians in training.^4,9,16^ The complex nature of these associations may contribute to remaining controversy about whether burnout and depression are discrete constructs vs gradations of the same underlying disorder,^17^ despite results of a recent meta-analysis reporting that burnout and depression are different constructs.^18^ Whether burnout increases risk of suicide after accounting for symptoms of depression is unclear; studies suggesting that burnout is associated with increased risk for suicidal ideation lack control for comorbid depression,^9,16,19^ and the few that control for it often use the Primary Care Evaluation of Mental Disorders (PRIME-MD),^4,9^ a 2-item screening tool that may not be an optimal measure of symptom severity or specificity.^20,21^ An analogous pattern exists between physician distress and patient care outcomes; both burnout and depression are associated with occupational consequences,^22,23,24,25,26^ including errors,^27^ in studies that often do not optimally account for both.^20,28,29^

Addressing physician well-being and reducing suicide risk requires understanding the associations between physician distress, including burnout and depression, and personal and professional outcomes. The primary objective of this study was to investigate whether burnout is associated with increased risk of suicidal ideation after accounting for concurrent symptoms of depression. The secondary objective of this study was to investigate whether depression and burnout are independently associated with self-reported medical errors. Preparatory to these aims was to explore divergent validity between burnout and depression assessment items using instruments frequently used to assess physician burnout and a validated measure^30,31,32^ of symptoms specific to depression (ie, not including nonspecific vegetative symptoms). This preparatory step provided evidence supporting the assumption that burnout and depression are distinct constructs in the data analyzed for this study, a necessary assumption of the study aims. We hypothesized that physician depression, but not burnout, would be directly associated with increased risk of suicidal ideation.

## Methods

### Study Population

This cross-sectional study used survey data collected between November 12, 2018, and February 15, 2019, from 1354 physicians practicing throughout the US. To assemble a representative convenience sample (≥1200 individuals), populations of 70% attending and 30% postgraduate trainee physicians of all age groups, sexes, and specialties were randomly sampled from the American Medical Association Physician Masterfile and invited in waves to participate. Participants were offered a small financial incentive to complete a confidential, voluntary electronic survey (including a prerequisite survey item obtaining informed consent). The study protocol was approved by the institutional review boards at Stanford University and the University of Illinois at Chicago. The study followed the Strengthening the Reporting of Observational Studies in Epidemiology (STROBE) reporting guideline for cross-sectional studies.^33^

### Measures

Burnout was assessed using subscales of 3 measures: the Stanford Professional Fulfillment Index (PFI),^34^ the Maslach Burnout Inventory–Human Services Survey for Medical Personnel (MBI),^35^ and the Mini-Z burnout survey.^36^ The PFI includes 2 subscales assessing burnout (4 work exhaustion items and 6 interpersonal disengagement items) that are scored using a 5-point Likert scale from “not at all” (0) to “extremely” (4).^34^ This measure has shown reliability, construct validity, and sensitivity to change among physicians.^34,37,38^ The MBI assesses analogous subscales (9 emotional exhaustion items and 5 depersonalization items) scored using a 7-point frequency-based Likert scale from “never”(0) to “every day” (6).^39^ Work exhaustion and interpersonal disengagement in the PFI are conceptually similar to emotional exhaustion and depersonalization in the MBI. The MBI is a measure of burnout with well-documented reliability and validity,^39,40,41,42^ including concurrent validity (correlations with occupational variables in expected directions among physicians).^22,23,24,43^ The Mini-Z burnout survey’s single item requests that respondents characterize their experience using their own definition of burnout; answers range in severity from “I enjoy my work. I have no symptoms of burnout” to “I feel completely burned out. I am at the point where I may need to seek help.”^36^ An endorsement of 1 of the 3 most severe response options including the word *burnout* is deemed to represent an individual experiencing burnout. Previous studies have repeatedly shown that this measure is associated with emotional exhaustion but does not assess depersonalization.^44,45,46^

Depression was assessed using the Patient-Reported Outcomes Measurement Information System (PROMIS) depression 4-item Short Form, which assesses symptoms specific to depression (ie, does not include items assessing vegetative symptoms) using a 5-point frequency-based Likert scale from never (1) to always (5) for scoring.^30,31,32^ It has demonstrated comparability to legacy measures,^47,48^ reliability, 31 content validity,^31,49^ concurrent validity,^30,50^ sensitivity to change,^48^ and capacity to assess symptom severity of clinical depression.^31,32,51^

Suicidal ideation was assessed using the question “During the past 12 months, have you had thoughts of taking your own life?”^52^ Those who responded yes were encouraged to reach out to their physician or the National Suicide Prevention Lifeline for assistance. The wording of this item and conceptual similarity with the National Comorbidity Survey item that assesses suicidal ideation facilitate comparison with nonphysician samples^52,53^ and suggest intuitive face validity. In addition, correlations with distress variables^4,9,26,54,55^ suggest concurrent validity. To our knowledge, evidence of other aspects of validity and reliability of this suicidal assessment item has not been published. Use of this item facilitates comparison of results of the present study with results of previous studies that have used the same assessment of suicidal ideation among physicians and physicians in training.^4,9,15,56,57^

Self-reported medical errors were assessed using a 4-item measure (eTable in the Supplement) developed in consultation with medical risk-management experts familiar with medical error.^34^ Used in previous research in postgraduate-trainee and attending-physician populations,^25,34^ this measure requests that respondents characterize their most recent experience with clinically significant errors, such as “I made a medical error that did result in patient harm” and “I ordered the wrong medication,” using a 6-point frequency-based Likert scale ranging from in the past week (5) to never (0).^34^ Scores are correlated with burnout and inversely correlated with professional fulfillment among physicians.^34^ This measure is designed to assess a broad range of errors and error rates and has intuitive face validity^34^ and conceptual similarity to other measures that assess this construct among physicians.^23,26,58,59^

### Statistical Analysis

To preliminarily explore divergent validity between burnout and depression, we used principal components analysis (PCA) with direct oblimin rotation. Two separate PCAs were conducted: (1) emotional exhaustion measures (MBI–emotional exhaustion, PFI–work exhaustion, and Mini-Z) and the depression measure and (2) depersonalization measures (MBI-depersonalization and PFI–interpersonal disengagement) and the depression measure. We defined an item as demonstrating divergent validity if it had a component loading of 0.30 or greater on 1 but not both components. This minimal (rather than higher) cut point threshold is a better indicator of divergent validity and unlikely to be shown in constructs with substantial degrees of overlap. The Cronbach α internal consistency was calculated from the current study data for all scales. Statistical analyses were conducted using IBM SPSS statistical software, version 25 (IBM).

Multivariate logistic regression models were specified to assess the association of burnout with odds of suicidal ideation. Burnout scale scores from the MBI, PFI, and Mini-Z were standardized to assess the association with suicidal ideation of each SD-unit increase in scale score for each instrument. The first model was unadjusted, the second was adjusted for depression, and the third was adjusted for depression and demographic characteristics (sex, race/ethnicity, training status, and age category).

Models were also specified to assess the association of depression with odds of self-reported medical error using a dichotomous variable reflecting high vs low rates of recent error^34^ in which scoring in the upper half of the overall score distribution (≥4) was high, corresponding to an average response of “within the past year” across all items.^34^ The first model was unadjusted, the second was adjusted for overall burnout (assessed by the PFI because this measure assesses overall burnout including a combination of work exhaustion and interpersonal disengagement), and the third was adjusted for burnout and demographic characteristics.

## Results

Of 1355 survey participants (11.4% of 11 884 invited), 1354 physicians completed the suicidal ideation measure and were included in this analysis; 893 (66.0%) were White, 1268 (93.6%) were non-Hispanic, 762 (56.3%) were men, 912 (67.4%) were non–primary care physicians, 934 (69.0%) were attending physicians, and 824 (60.9%) were younger than 45 years. Modal category characteristics were White race/ethnicity, male sex, and non–primary care physicians younger than 45 years. Table 1 gives demographic (sex, age category) and occupational (training status, practice type, and specialty) characteristics of participants and nonresponders (invited physicians who elected not to participate).

**Table 1.** Characteristics of Survey Participants and Nonresponders

Characteristic	No. (%)	
	Participants (n = 1354)	Nonresponders (n = 10 529)^a^
Sex		
Female	579 (42.8)	3678 (34.9)
Male	762 (56.3)	6833 (64.9)
Missing	13 (1.0)	18 (0.2)
Age, y		
<35	439 (32.4)	1854 (17.6)
35-44	385 (28.4)	2245 (21.3)
45-54	243 (18.0)	2444 (23.2)
55-64	193 (14.3)	2291 (21.8)
≥65	94 (6.9)	1688 (16.0)
Training status		
Attending physician	934 (69.0)	9059 (86.0)
Trainee (resident or fellow)	420 (31.0)	1470 (14.0)
Practice type		
Nongovernment hospital	473 (34.9)	1882 (17.9)
Group practice	404 (29.8)	4018 (38.2)
Government hospital (city, county, state, or federal)	132 (9.8)	1166 (11.1)
Small private practice^b^	114 (8.4)	1777 (16.9)
Missing or other practice type^c^	231 (17.1)	1686 (16.0)
Specialty		
Anesthesiology	96 (7.1)	550 (5.2)
Dermatology	24 (1.8)	209 (2.0)
Emergency medicine	74 (5.5)	525 (5.0)
Family medicine	167 (12.3)	1290 (12.3)
Internal medicine	184 (13.6)	1472 (14.0)
Internal medicine subspecialty	127 (9.4)	1468 (13.9)
Neurology	28 (2.1)	207 (2.0)
Obstetrics and gynecology	96 (7.1)	623 (5.9)
Ophthalmology	30 (2.2)	256 (2.4)
Pathology	4 (0.3)	43 (0.4)
Pediatrics	91 (6.7)	610 (5.8)
Pediatrics subspecialty	63 (4.7)	318 (3.0)
Physical medicine	13 (1.0)	108 (1.0)
Psychiatry	90 (6.7)	629 (6.0)
Radiology	53 (3.9)	491 (4.7)
Surgery	62 (4.6)	390 (3.7)
General surgery subspecialty	71 (5.2)	714 (6.8)
Other	81 (6.0)	626 (5.9)

^a^ Nonresponder counts represent the population invited with elimination of the undeliverable and duplicate emails identified.

^b^ Small private practices include self-employed solo practices and full or part owners of a 2-physician practice.

^c^ Missing or other practice type includes no classification, medical school, other patient care, health maintenance organization, and locum tenens.

All scales had acceptable to excellent internal consistency reliability. Reliability coefficients for emotional exhaustion (α = 0.93) and depersonalization (α = 0.84) in the MBI were similar to those previously reported.^60^ Reliability coefficients for work exhaustion (α = 0.87), interpersonal disengagement (α = 0.92), and overall burnout (α = 0.93) in the PFI were also similar to those previously reported.^34^ The PROMIS depression scale and self-reported medical error scale had excellent (α = 0.91) and acceptable (α = 0.76) internal consistency reliability, respectively.

### Distinguishing Burnout From Depression

Table 2 shows factor loadings for burnout and depression assessment items from PCAs with direct oblimin rotation in which values of 0.30 or above are high and values less than 0.30 are low. The PCAs showed divergent validity between and respective convergent validity within depression and all 25 items from the 3 burnout instruments, with the exception of 1 MBI–emotional exhaustion item worded “I’m at the end of my rope.” This item had approximately equal component loadings on both the emotional exhaustion and work exhaustion subscale component (0.46) and the depression component (0.40). All 13 other emotional exhaustion subscale items in the PCA (PFI–work exhaustion, MBI–emotional exhaustion, and Mini-Z) loaded high on the burnout component (range, 0.49-0.96) and low on the depression component (range, –0.20 to 0.24). In a subsequent PCA of the interpersonal disengagement and depersonalization subscale of burnout and depression, all 11 PFI–interpersonal disengagement and MBI–depersonalization assessment items showed high loadings on the burnout component (range, 0.58-0.90) and low loadings on the depression component (range, –0.09 to 0.27). In both PCAs, all PROMIS depression items loaded low on the burnout subscale components (range, –0.06 to 0.17) and high on the depression component (range, 0.74-0.93).

**Table 2.** Factor Loadings of Burnout and Depression Assessment Items

Measure	Item wording	Pattern coefficient^a^	
		Burnout	Depression
Work exhaustion (Stanford Professional Fulfillment Index)	“A sense of dread when I think about work I have to do”	0.67^b^	0.17
	“Physically exhausted at work”	0.72^b^	0.02
	“Lacking in enthusiasm at work”	0.65^b^	0.15
	“Emotionally exhausted at work”	0.77^b^	0.08
Emotional exhaustion (Maslach Burnout Inventory)	“I feel emotionally drained from my work”	0.92^b^	−0.09
	“I feel used up at the end of the workday”	0.96^b^	−0.20
	“I feel fatigued when I get up in the morning and have to face another day on the job”	0.83^b^	−0.02
	“Working with people all day is really a strain for me”	0.62^b^	0.13
	“I feel burned out from my work”	0.88^b^	0.02
	“I feel frustrated by my job”	0.86^b^	−0.02
	“I feel I’m working too hard on my job”	0.86^b^	−0.09
	“Working with people directly puts too much stress on me”	0.49^b^	0.24
	“I feel like I’m at the end of my rope”	0.46^b^	0.40^c^
Burnout survey (Mini-Z)	“Using your own definition of ‘burnout,’ please choose one of the options below”	0.75^b^	0.12
Depression (NIH PROMIS Short Form)	“I felt worthless”	−0.06	0.90^c^
	“I felt helpless”	0.06	0.86^c^
	“I felt depressed”	0.17	0.74^c^
	“I felt hopeless”	0.01	0.91^c^
Interpersonal disengagement (Stanford Professional Fulfillment Index)	“Less empathetic with my patients”	0.89^b^	−0.09
	“Less empathetic with my colleagues”	0.67^b^	0.15
	“Less sensitive to others’ feelings/emotions”	0.82^b^	0.01
	“Less interested in talking with my patients”	0.87^b^	−0.06
	“Less connected with my patients”	0.90^b^	−0.08
	“Less connected with my colleagues”	0.58^b^	0.27
Depersonalization (Maslach Burnout Inventory)	“I feel I treat some patients as if they were impersonal objects”	0.77^b^	−0.05
	“I’ve become more callous toward people since I took this job”	0.79^b^	0.05
	“I worry that this job is hardening me emotionally”	0.72^b^	0.16
	“I don’t really care what happens to some patients”	0.63^b^	−0.03
	“I feel patients blame me for some of their problems”	0.60^b^	0.00
Depression (NIH PROMIS Short Form)	“I felt worthless”	−0.02	0.87^c^
	“I felt helpless”	0.00	0.90^c^
	“I felt depressed”	0.10	0.81^c^
	“I felt hopeless”	−0.02	0.93^c^

Abbreviations: NIH, National Institutes of Health; PROMIS, Patient-Reported Outcomes Measurement Information System.

^a^ Principal component analysis with direct oblimin rotation, pattern matrix loadings. Pattern coefficients 0.30 or greater are high, and those less than 0.30 are low.

^b^ Item loaded moderately to highly into burnout component.

^c^ Item loaded moderately to highly into depression component.

### Associations of Burnout and Depression With Suicidal Ideation

In aggregate, 75 of 1354 physicians (5.5%) reported having thoughts of taking their own life in the previous 12 months. The prevalence of suicidal ideation by participant characteristics, including age, race/ethnicity, sex, practice type, and specialty, is shown in Table 3.

**Table 3.** Prevalence of Suicidal Ideation by Participant Characteristics

Characteristic	Participants, No. (%) (N = 1354)		
	No suicidal ideation	Suicidal ideation	Total
Sex			
Female	537 (39.7)	42 (3.1)	579 (42.8)
Male	730 (53.9)	32 (2.4)	762 (56.3)
Missing	12 (0.9)	1 (0.1)	13 (1.0)
Age, y			
<35	418 (30.9)	21 (1.6)	439 (32.4)
35-44	360 (26.6)	25 (1.9)	385 (28.4)
45-54	227 (16.8)	16 (1.2)	243 (18.0)
55-64	183 (13.5)	10 (0.7)	193 (14.3)
≥65	91 (6.7)	3 (0.2)	94 (6.9)
Race			
Asian	286 (21.1)	6 (0.4)	292 (21.6)
Black or African American	50 (3.7)	4 (0.3)	54 (4.0)
White	832 (61.5)	61 (4.5)	893 (66.0)
Other or missing	98 (7.2)	2 (0.1)	100 (7.4)
Ethnicity			
Hispanic	65 (4.8)	7 (0.5)	72 (5.3)
Non-Hispanic	1200 (88.6)	68 (5.0)	1268 (93.6)
Missing	14 (1.0)	0	14 (1.0)
Training status			
Attending physician	881 (65.1)	53 (3.9)	934 (69.0)
Trainee (resident or fellow)	398 (29.4)	22 (1.6)	420 (31.0)
Practice type			
Nongovernment hospital	451 (33.3)	22 (1.6)	473 (34.9)
Group practice	379 (28.0)	25 (1.9)	404 (29.8)
Government hospital (city, county, state, or federal)	125 (9.2)	7 (0.5)	132 (9.8)
Small private practice^a^	110 (8.1)	4 (0.3)	114 (8.4)
Missing or other practice type^b^	214 (15.8)	17 (1.3)	231 (17.1)
Specialty			
Anesthesiology	91 (6.7)	5 (0.4)	96 (7.1)
Dermatology	22 (1.6)	2 (0.2)	24 (1.8)
Emergency medicine	71 (5.2)	3 (0.2)	74 (5.5)
Family medicine	157 (11.6)	10 (0.7)	167 (12.3)
Internal medicine	178 (13.2)	6 (0.4)	184 (13.6)
Internal medicine subspecialty	123 (9.1)	4 (0.3)	127 (9.4)
Neurology	27 (2.0)	1 (0.07)	28 (2.1)
Obstetrics and gynecology	90 (6.7)	6 (0.4)	96 (7.1)
Ophthalmology	29 (2.1)	1 (0.07)	30 (2.2)
Pathology	3 (0.2)	1 (0.07)	4 (0.3)
Pediatrics	88 (6.5)	3 (0.2)	91 (6.7)
Pediatrics subspecialty	58 (4.3)	5 (0.4)	63 (4.7)
Physical medicine	12 (0.9)	1 (0.07)	13 (1.0)
Psychiatry	82 (6.1)	8 (0.6)	90 (6.7)
Radiology	52 (3.8)	1 (0.07)	53 (3.9)
Surgery	56 (4.1)	6 (0.4)	62 (4.6)
General surgery subspecialty	67 (5.0)	4 (0.3)	71 (5.2)
Other	73 (5.4)	8 (0.6)	81 (6.0)

^a^ Small private practices include self-employed solo practices (n = 97) and full or part owners of a 2-physician practice (n = 17).

^b^ Missing or other practice type includes no classification (n = 200), medical school (n = 14), other patient care (n = 10), health maintenance organization (n = 4), and locum tenens (n = 3).

The logistic regression model revealed an association of burnout scores and burnout subscale scores with suicidal ideation before and after adjusting for depression and demographic variables (Table 4). Each increase of 1 SD in score on the PFI overall burnout scale was associated with 85% greater odds of experiencing suicidal ideation (odds ratio [OR],  1.85; 95% CI, 1.47-2.31) before adjusting for depression. After adjusting for depression, higher PFI overall burnout scores were not associated with greater risk of suicidal ideation (OR, 0.85; 95% CI, 0.63-1.17). This same pattern of results was consistent with all burnout subscale scores (PFI–work exhaustion, PFI–interpersonal disengagement, MBI–emotional exhaustion, and MBI–depersonalization); each was associated with greater risk of suicidal ideation before but not after adjusting for depression. After adjusting for overall burnout (PFI), sex, race/ethnicity, training status, and age category, each increase of 1 SD in score on the PROMIS depression scale was associated with 202% greater odds of suicidal ideation (OR, 3.02; 95% CI, 2.30-3.95).

**Table 4.** Logistic Regression Modeling of the Association Between Burnout and Suicidal Ideation

Variable	Odds ratio (95% CI)		
	Model 1^a^	Model 2^b^	Model 3^c^
Burnout (Stanford Professional Fulfillment Index)	1.85 (1.47-2.31)	0.85 (0.63-1.17)	0.88 (0.64-1.22)
Work exhaustion (Stanford Professional Fulfillment Index)	1.92 (1.52-2.41)	0.85 (0.62-1.16)	0.83 (0.60-1.15)
Interpersonal disengagement (Stanford Professional Fulfillment Index)	1.66 (1.33-2.07)	0.89 (0.67-1.18)	0.94 (0.70-1.26)
Emotional exhaustion (Maslach Burnout Inventory)	2.16 (1.69-2.75)	1.03 (0.75-1.41)	0.94 (0.68-1.31)
Depersonalization (Maslach Burnout Inventory)	1.82 (1.48-2.25)	1.08 (0.84-1.40)	1.12 (0.86-1.46)

^a^ Model 1 was unadjusted.

^b^ Model 2 was adjusted for depression.

^c^ Model 3 was adjusted for depression, sex, race/ethnicity, training status, and age category.

### Associations of Burnout and Depression With Self-reported Medical Errors

Before adjusting for overall burnout, each SD-unit increase in depression score was associated with 27% greater odds of increased self-reported medical errors (OR, 1.27; 95% CI, 1.14-1.43). After adjusting for burnout using the PFI, higher depression scores were not associated with greater odds of self-reported medical errors (OR, 1.01; 95% CI, 0.88-1.16); this result was consistent with that of the final model, which also adjusted for sex, race/ethnicity, training status, and age category (OR, 1.06; 95% CI, 0.91-1.22). In models adjusted for the same variables, overall burnout was associated with 44% to 48% greater odds of increased self-reported medical errors (OR in the model adjusted for depression, 1.48; 95% CI, 1.28-1.71; OR in the model adjusted for depression, sex, race/ethnicity, training status, and age, 1.44; 95% CI, 1.24-1.67).

## Discussion

The results of this cross-sectional study suggest that burnout was associated with suicidal ideation in physicians before but not after adjusting for depression and that depression was associated with suicidal ideation after adjustment for burnout. The results showed an opposite pattern of associations with self-reported medical errors; burnout but not depression was associated with self-reported medical errors in a fully adjusted model. In addition, PCA preparatory to analyses addressing the primary study aim provided additional evidence that burnout and depression are different constructs, with all but 1 of 25 assessment items showing associated divergent validity. The association this study showed between depression and suicidal ideation in physicians is consistent with research in the general population.^61^ Common factors associated with suicide or suicidal ideation in the general population include depression, hopelessness, impairment, and previous suicide attempts.^62^

Depression is associated with serious medical morbidity, whereas the association of burnout with suicidal ideation is either the result of confounding effects with comorbid depression or is indirect to the degree that burnout is associated with depression.^4,9,61^ Previously observed associations between burnout and suicidal ideation^4,9,19^ may have resulted from failure to adjust for depression or from adjustment for depression using a screening instrument (ie, the PRIME-MD^4,9^) that does not optimally capture symptom severity or specificity.^20,21^ The absence of a direct association of burnout with suicidal ideation is also consistent with the World Health Organization’s definitions of these constructs: burnout is an occupational distress syndrome rather than a clinical psychiatric diagnosis indicating distress in multiple life domains.^13^

There is also biologically plausible evidence of the distinction between occupational distress (burnout) and depression. For example, burnout and depression are differentially associated with concentrations of the microinflammation biomarkers high-sensitivity C-reactive protein and fibrinogen.^63^ Furthermore, the glucocorticoid receptor and brain-derived neurotrophic factor genes display different methylation patterns during depression than during occupational stress.^64^ Depression is a serious medical disorder, responsible for more morbidity worldwide than any other medical problem and warranting medical evaluation and evidence-based clinical treatment (eg, pharmacologic or psychotherapy).^18^ Burnout, in contrast, is a serious occupational distress syndrome affecting patient care^25,29,65^ and is directly associated with self-reported medical error. The syndrome is frequently associated with characteristics of the work environment (excessive demands and inadequate resources) and is ideally addressed through system-based occupational interventions.

Occupational burnout also has important health implications and is associated with increases in insomnia,^66^ mental illness symptoms,^66^ headaches,^66^ severe injury,^66^ type 2 diabetes,^67^ extended fatigue,^66^ coronary heart disease,^63^ gastrointestinal and respiratory concerns,^66^ myocardial infarction, atrial fibrillation,^68^ musculoskeletal discomfort,^63,66^ and all-cause mortality.^66^ System-level efforts should be pursued to mitigate burnout to reduce these risks. Analogous to depression, however, each of these health consequences is distinct from burnout and, once present, requires specific treatment. Burnout may be associated with cortisol dysregulation,^69^ hypercholesterolemia,^70^ decreased fibrinolytic capacity,^71^ and telomere function,^72^ although the mechanisms by which burnout may contribute to those conditions have not yet been elucidated. Similar to the association between burnout and suicidal ideation observed in this study, the association between burnout and some of these conditions may not be direct. Although burnout is not a clinical diagnosis, it may be more important than depression in terms of occupational consequences,^25,28,29,59^ consistent with the current study finding that burnout was directly associated with an increased risk of self-reported medical errors. Furthermore, physician burnout has been shown to be associated with unsolicited patient complaints, whereas in the same study, depression was not.^25^

Although some researchers continue to dispute the independence of burnout and depression, psychometric studies have predominantly shown that these 2 constructs are distinct, including a recent systematic review and meta-analysis of 67 studies that showed that burnout differed from depression.^18^ The results of the current study are consistent with the premise that burnout differs from depression. The single burnout assessment item that did not show divergent validity from depression assessment items was an MBI–emotional exhaustion item with wording that intuitively seems inclusive of more generalized depression symptoms than occupation-specific distress.

### Limitations

This study has limitations. All of the measures were self-reported and may thus be subject to participant bias. Furthermore, although the associations between the domains assessed are unlikely to differ, it is possible that the point prevalence of burnout, depression, suicidal ideation, and self-reported medical error rate in the convenience sample, which had a low response rate (common among surveys of health care professionals^73^), does not generalize to US physicians at large. However, previous research using the same methods (including sensitive questions) among US physicians nationwide followed by survey of a random sample of nonrespondents showed reasonable equivalence between respondents and nonrespondents.^15,57^ Only 75 physicians in the sample reported suicidal ideation, which is inadequate for separate subgroup analyses of attending physicians and residents. In addition, these cross-sectional associations do not indicate causality. Future investigations are warranted to evaluate causal relationships between burnout, depression, suicidal ideation, and medical error.

## Conclusions

The findings of this cross-sectional study suggest that depression but not burnout is directly associated with greater suicidal ideation in physicians. In addition, the results suggest that burnout, not depression, is directly associated with an increased risk of self-reported medical errors. The findings of this study suggest that burnout without depression does not increase suicide risk and can therefore be safely addressed outside of mental health care.

## Notes

Supplement. **eTable.** Self-reported medical errors
