# Error in the Methods Section

**DOI:** 10.1001/jamanetworkopen.2021.15436

**Published:** 2021-05-26

In the Original Investigation titled “Association of Physician Burnout With Suicidal Ideation and Medical Errors,”^1^ published December 9, 2020, in the last paragraph of the Statistical Analysis subsection of the Methods section, “corresponding to an average response of ‘within the past year’ across all items” was changed to “A score of 4 corresponded to a response of ‘within my lifetime’ across all items or a more recent error in at least 1 category.” This article has been corrected.^1^
