# Supplementary material for: Association of Physician Burnout With Suicidal Ideation and Medical Errors
Source: JAMA Netw Open. 2020 Dec 9;3(12):e2028780. doi: 10.1001/jamanetworkopen.2020.28780 (PMC7726631; doi:10.1001/jamanetworkopen.2020.28780)
Supplement: Supplement. — eTable. Self-reported medical errors [file jamanetwopen-e2028780-s001.pdf]

Supplemental Online Content

Menon NK, Shanafelt TD, Sinsky CA, et al. Association of physician burnout with suicidal ideation and medical errors. *JAMA Netw Open*. 2020;3(12):e2028780. doi:10.1001/jamanetworkopen.2020.28780

eTable. Self-reported medical errors

This supplementary material has been provided by the authors to give readers additional information about their work.

© 2020 Menon et al. *JAMA Network Open*.

eTable. Self-reported medical errors **(By Mickey Trockel, MD, PhD, Stanford University)**

Please indicate the most recent time you experienced each of the following things:

	**In the last week**	**In the last month**	**In the last 3 months**	**In the last year**	**In my lifetime**	**Never**
	**5**	**4**	**3**	**2**	**1**	**0**
I made a major medical error that could have resulted in patient harm	**[ ]**	**[ ]**	**[ ]**	**[ ]**	**[ ]**	**[ ]**
I made a medical error that did result in patient harm	**[ ]**	**[ ]**	**[ ]**	**[ ]**	**[ ]**	**[ ]**
I ordered the wrong medication	**[ ]**	**[ ]**	**[ ]**	**[ ]**	**[ ]**	**[ ]**
I ordered the wrong lab test	**[ ]**	**[ ]**	**[ ]**	**[ ]**	**[ ]**	**[ ]**

© 2020 Menon et al. *JAMA Network Open*.
